# Right heart thrombus in transit: a series of two cases

**DOI:** 10.1186/s13089-017-0069-9

**Published:** 2017-06-15

**Authors:** Eva Otoupalova, Bhavinkumar Dalal, Brian Renard

**Affiliations:** 1 Department of Internal Medicine, Oakland University William Beaumont School of Medicine, 3601 W 13 Mile Rd., Royal Oak, MI 48073 USA; 2 Department of Pulmonary and Critical Care, Oakland University William Beaumont School of Medicine, 3601 W 13 Mile Rd., Royal Oak, MI 48073 USA; 3 Department of Cardiology, Oakland University William Beaumont School of Medicine, 3601 W 13 Mile Rd., Royal Oak, MI 48073 USA

## Abstract

Right heart thrombus in transit is an increasingly recognized medical emergency with very high mortality rate. Echocardiography helps to establish the diagnosis and can differentiate between right heart thrombi that result from atrial fibrillation and those originating from deep venous thrombosis. We present two cases of right heart thrombus in transit diagnosed with echocardiography that were managed with different approaches.

**Electronic supplementary material:**

The online version of this article (doi:10.1186/s13089-017-0069-9) contains supplementary material, which is available to authorized users.

## Background

Increased use of echocardiography has led to greater detection of atrial thrombi in recent years. In absence of atrial fibrillation, right heart thrombi mostly represent an embolus in transit originating from a deep venous thrombosis. Right heart thrombi occur in about 4% [1] of cases of acute PE and have very high mortality rate. Treatment options include surgery, thrombolysis, and catheter-based interventions. We present two cases of right heart thrombus in transit diagnosed by echocardiography.

## Case 1

79-year-old female with history of hypertension presented with acute abdominal pain for 1 h. She was hypotensive in the emergency room. Initial ECG showed inferior Q-waves and ST depressions in the lateral leads. Right-sided ECG revealed 1.5 mm ST elevation in right-sided lead V4 (RV4). Laboratory workup was significant for troponin elevation 0.14 ng/mL. Emergent heart catheterization was done and revealed non-obstructive moderate coronary artery disease.

**Fig. 1:**
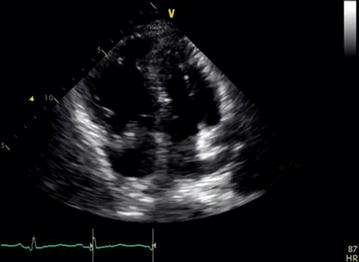
Apical 4-chamber view with a large mobile thrombus in the right atrium crossing into the right ventricle. The right ventricle is severely enlarged

**Fig. 2:**
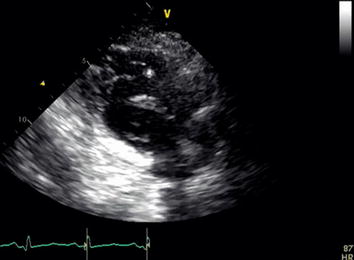
Right ventricular inflow view with large mobile thrombus crossing into the right ventricle

An urgent bedside transthoracic echocardiography (TTE) was performed and revealed large, free-floating thrombus in right atrium, septal flattening, and severely decreased right ventricular systolic function (Figs. 1, 2; Additional files 1, 2). Right ventricular systolic pressure (RVSP) elevated at 56 mmHg.

Patient was taken to surgery and underwent atriotomy and pulmonary arteriotomy on cardiopulmonary bypass. Despite successful surgery, patient developed infectious complications and passed 5 days later.

## Case 2

**Fig. 3:**
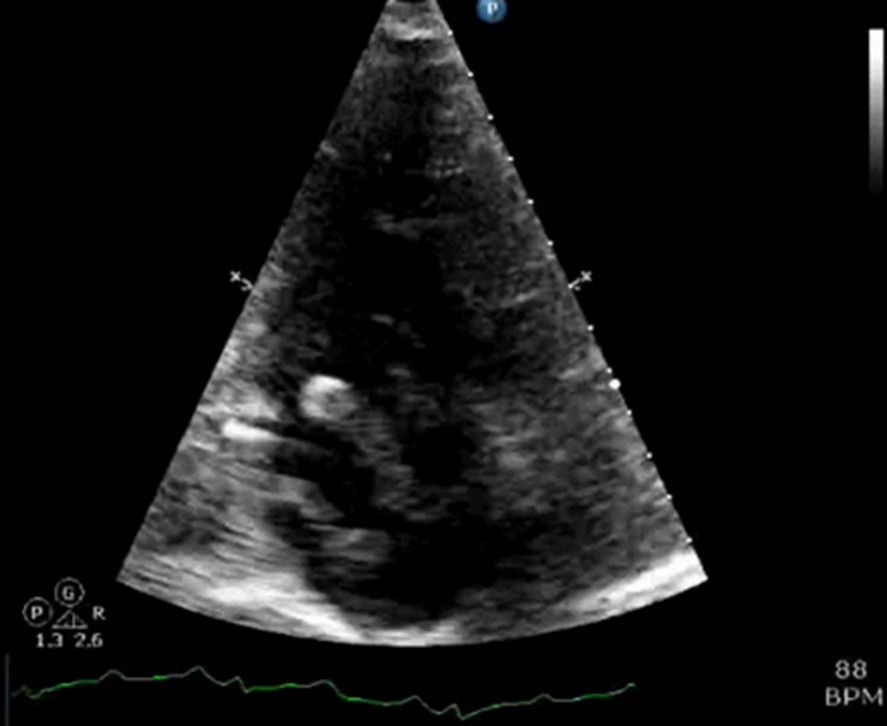
Apical 4-chamber view with a large mobile thrombus in the right atrium crossing into the right ventricle. There is flattening of the interventricular septum consistent with right ventricular pressure overload. Mc Connell’s sign is present

**Fig. 4:**
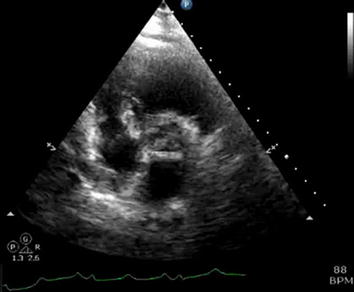
Parasternal short axis view of large mobile thrombus in the right atrium

89-year-old female presented with dyspnea for 1 day. In the emergency room, she was normotensive but tachycardic and tachypneic. Initial ECG revealed normal sinus rhythm, right bundle brunch block, and T wave inversion in V1–V3. Initial laboratory workup showed elevated troponin 2.60 ng/mL. TTE was performed and showed mobile thrombus in the right atrium and severely enlarged right ventricle with RVSP 45–50 mmHg (Figs. 3, 4; Additional files 3, 4). Akinesis of mid-free wall of the right ventricle and normal motion of apex consistent with Mc Connell’s sign was present.

Patient was taken to cardiac catheterization laboratory to undergo catheter directed AngioVac thrombus extraction. However, upon obtaining access, patient became hypotensive and bradycardic. A transesophageal echocardiography demonstrated markedly worsened dilation of the right ventricle with a very small left ventricle indicative of severe right heart strain. The right atrial thrombus was still visible and appeared unchanged. Resuscitative measures were undertaken; unfortunately, the patient did not respond and passed.

## Discussion and literature review

Both of our patients died; however, our first patient died due to infectious complications and not due to obstructive shock itself. As appropriately described in literature, our first patient had surgical embolectomy and survived from acute problem of right heart thrombus (RHT), while in the second case, there was not enough time before patient deteriorated and died. This feature describes the importance of early diagnosis and aggressive treatment of RHT. In current era, many bedside echocardiographs are performed not only by cardiology team but also by emergency and internal medicine house staff; recognition of RHT and its aggressive treatment is crucial.

Right heart thrombi occur in about 4% of pulmonary embolism (PE) [1]. They result either from dislodging clot from deep venous thrombosis, or form in situ mostly due to atrial fibrillation. They can also be associated with intracardiac foreign bodies such as pacemakers and prosthetic valves.

Mortality rate in patients with RHT is about 28%, with mortality rates in untreated patients of 80–100%. In contrast, the in-hospital mortality rate for acute pulmonary embolism (PE) is about 2.5% [1]. Patients with RHT have shorter duration of symptoms, lower systolic blood pressure, more frequent right ventricular hypokinesis and congestive heart failure [2].

Three major types of right heart thrombus can be distinguished on echocardiography [2]: type A is most common, is usually the result of deep venous thrombosis and has the highest risk of embolization. It is elongated, with a worm-like appearance and is freely mobile within the heart chambers. Type B thrombus is thought to originate within the atrium or ventricle, and is firmly attached to the chamber wall and ovoid shape. Type C thrombi are rare, highly mobile and resembling cardiac myxomas. Transesophageal echocardiography (TEE) provides better visualization of the thrombus and should be considered when TTE is suboptimal or non-diagnostic [3]; it can localize thrombus in pulmonary artery or lodged in patent foramen ovale.

Both of our patients had type A thrombus. In addition, both of our patients had signs of severe right ventricular pressure overload. Akinesis of mid-free wall of the right ventricle and normal motion of apex such as in our second patient (McConnell’s sign) is particularly suggestive of PE [4].

Treatment modalities for RHT include anticoagulation therapy, systemic thrombolysis and surgical embolectomy. Optimal therapeutic approach is still subject of discussion as no randomized controlled trials have directly compared the treatment methods. In a meta-analysis of 119 patients by Kinney from 1989, there was a small survival benefit with anticoagulation (70%) when compared to surgical embolectomy or thrombolysis (62%) [5]. However, not all patients had a pulmonary embolism in the study. A multicenter observational European study from the same year reported a markedly lower mortality with surgical embolectomy (27%) as compared to anticoagulation (>60%) [2]. A meta-analysis by Rose from 2002 of 177 patients with RHT demonstrated a superiority of thrombolytic therapy over surgical embolectomy (OR for mortality: 2.83, 95% CI 1.04–7.69) and anticoagulation (OR for mortality: 3.03, 95% CI 1.02–3.125) [6]. These results were confirmed by the largest meta-analysis to date by Ganesh et al. from 2015 that evaluated total 328 patients. In this meta-analysis, 70 patients received anticoagulation, 122 patients received thrombolysis and 120 patients had surgical embolectomy. Mortality rates were 37.1, 18.3 and 13.9%, respectively. After adjusting for age and hemodynamic status, the OR for survival was 4.83 (95% CI 1.51–15.36) with thrombolysis and 2.61 (95% CI 0.90–7.58) with surgical embolectomy [7]. Data on catheter-based interventions are scarce.

## Conclusion

As our two cases illustrate, right heart clot in transit is a medical emergency with high mortality that requires immediate evaluation and treatment. TTE is preferred diagnostic method and helps distinguish between clot formed in situ and clot in transit. Thrombolysis and surgical embolectomy are superior to anticoagulation, with thrombolysis possibly offering a modest survival benefit.

## Additional files

**Additional file 1.** Apical 4-chamber view with a large mobile thrombus in the right atrium crossing into the right ventricle. The right ventricle is severely enlarged.

**Additional file 2.** Right ventricular inflow view with large mobile thrombus crossing into the right ventricle.

**Additional file 3.** Apical 4-chamber view with a large mobile thrombus in the right atrium crossing into the right ventricle. There is flattening of the interventricular septum consistent with right ventricular pressure overload. Mc Connell’s sign is present.

**Additional file 4.** Parasternal short axis view of large mobile thrombus in the right atrium.

## Abbreviations

ECG: electrocardiogram
TTE: transthoracic echocardiography
TEE: transesophageal echocardiography
RHT: right heart thrombus
RVSP: right ventricular systolic pressure
PE: pulmonary embolism
